# Supplementary material for: Effect of different running protocols on bone morphology and microarchitecture of the forelimbs in a male Wistar rat model
Source: PLoS One. 2024 Nov 7;19(11):e0308974. doi: 10.1371/journal.pone.0308974 (PMC11542884; doi:10.1371/journal.pone.0308974)
Supplement: S1 Table — Measurements are expressed as mean ± SD, measured by μCT (Bruker SkyScan 1176, Kontich, Belgium) and analyzed by DragonFly software (version 2022.2 Build 1399). SED: Sedentary group; HIIT: High Intensity Interval Training group; CR: Continuous Running group; ComR: Combined Running group. BV/TV: Bone Volume/Tissue Volume; Tb.N: Trabecular Number; Tb.Sp: Trabecular Spacing; Tb.Th: Trabecular Thickness. (PDF) [file pone.0308974.s001.pdf]

S 1 Table: Trabecular microarchitecture analysis by µCT of the radius as a function of running modality.

Trabecular microarchitectural parameters		SED	HIIT	CR	ComR
**Radius**	BV/TV (%)	82.3 ± 1.7	82.4 ± 2.6	81.5 ± 1.9	81.7 ± 1.7
	Tb.N (mm^{-1})	2.48 ± 0.20	2.49 ± 0.30	2.37 ± 0.24	2.32 ± 0.32
	Tb.Sp (mm)	0.28 ± 0.04	0.29 ± 0.05	0.30 ± 0.04	0.31 ± 0.06
	Tb.Th (mm)	0.124 ± 0.015	0.120 ± 0.012	0.125 ± 0.008	0.128 ± .008
**Radius proximal**	BV/TV (%)	85.5 ± 2.5	87.1 ± 1.5	85.7 ± 2.5	87.4 ± 1.9
	Tb.N (mm^{-1})	4.25 ± 0.30	4.20 ± 0.61	4.31 ± 0.41	4.42 ± 0.25
	Tb.Sp (mm)	0.115 ± 0.009	0.125 ± 0.036	0.115 ± 0.022	0.107 ± 0.016
	Tb.Th (mm)	0.121 ± 0.015	0.118 ± 0.007	0.118 ± 0.004	0.120 ± 0.008
**Radius distal**	BV/TV (%)	61.0 ± 5.0	60.0 ± 5.0	61.0 ± 6.0	61.0 ± 3.0
	Tb.N (mm^{-1})	4.89 ± 0.25	4.93 ± 0.49	4.84 ± 0.22	4.98 ± 0.15
	Tb.Sp (mm)	0.120 ± 0.010	0.121 ± 0.015	0.121 ± 0.012	0.114 ± 0.006
	Tb.Th (mm)	0.086 ± 0.006	0.084 ± 0.008	0.086 ± 0.006	0.087 ± 0.004
