# Supplementary material for: Effect of different running protocols on bone morphology and microarchitecture of the forelimbs in a male Wistar rat model
Source: PLoS One. 2024 Nov 7;19(11):e0308974. doi: 10.1371/journal.pone.0308974 (PMC11542884; doi:10.1371/journal.pone.0308974)
Supplement: S2 Table — Measurements are expressed as mean ± SD, measured by μCT (Bruker SkyScan 1176, Kontich, Belgium) and analyzed by DragonFly software (version 2022.2 Build 1399). S: p-value <0.05 vs SED; C: p-value <0.05 vs CR; I: p-value < 0.05 vs HIIT. SED: Sedentary group; HIIT: High Intensity Interval Training group; CR: Continuous Running group; ComR: Combined Running group. BV/TV: Bone Volume/Tissue Volume; Tb.N: Trabecular Number; Tb.Sp: Trabecular Spacing; Tb.Th: Trabecular Thickness. (PDF) [file pone.0308974.s002.pdf]

S 2 Table: Trabecular microarchitecture analysis by µCT of the ulna as a function of running modality.

Microarchitectural trabecular parameters		SED	HIIT	CR	ComR
**Ulna**	BV.TV (%)	82.5 ± 1.0	83.6 ± 1.8	82.3 ± 1.4	82.6 ± 1.5
	Tb.N (mm^{-1})	3.03 ± 0.20	3.15 ± 0.20	3.03 ± 0.27	3.00 ± 0.19
	Tb.Sp (mm)	0.194 ± 0.020	0.185 ± 0.017	0.196 ± 0.026	0.194 ± 0.022
	Tb.Th (mm)	0.137 ± 0.008	0.133 ± 0.010	0.136 ± 0.007	0.140 ± 0.005
**Ulna proximal**	BV.TV	63.1 ± 3.3	65.8 ± 3.9	64.3 ± 2.1	63.7 ± 2.5
	Tb.N (mm^{-1})	4.27 ± 0.17	4.31 ± 0.18	3.88 ± 1.46	4.41 ± 0.20
	Tb.Sp (mm)	0.142 ± 0.011	0.137 ± 0.006	0.130 ± 0.015	0.134 ± 0.010
	Tb.Th (mm)	0.093 ± 0.004	0.096 ± 0.008	0.084 ± 0.032	0.094 ± 0.004
**Ulna distal**	BV.TV (%)	65.0 ± 2.8	**57.6 ± 5.9 S**	**59.6 ± 4.6 S**	**65.1 ± 2.6 C I**
	Tb.N (mm^{-1})	4.90 ± 0.29	4.98 ± 0.46	5.08 ± 0.29	4.96 ± 0.28
	Tb.Sp (mm)	0.120 ± 0.012	0.124 ± 0.014	0.118 ± 0.011	0.115 ± 0.011
	Tb.Th (mm)	0.084 ± 0.004	0.079 ± 0.009	0.071 ± 0.027	**0.088 ± 0.005 C I**
