# Supplementary material for: Effect of different running protocols on bone morphology and microarchitecture of the forelimbs in a male Wistar rat model
Source: PLoS One. 2024 Nov 7;19(11):e0308974. doi: 10.1371/journal.pone.0308974 (PMC11542884; doi:10.1371/journal.pone.0308974)
Supplement: S3 Table — Measurements are expressed as mean ± SD, measured by μCT (Bruker SkyScan 1176, Kontich, Belgium) and analyzed by DragonFly software (version 2022.2 Build 1399). SED: Sedentary group; HIIT: High Intensity Interval Training group; CR: Continuous Running group; ComR: Combined Running group. BV/TV: Bone Volume/Tissue Volume; Tb.N: Trabecular Number; Tb.Sp: Trabecular Spacing; Tb.Th: Trabecular Thickness. (PDF) [file pone.0308974.s003.pdf]

S 3 Table: Trabecular microarchitectural analysis by µCT of the humerus as a function of running modality.

Microarchitectural trabecular parameters		SED	HIIT	CR	ComR
**Humerus**	BV/TV (%)	63.0 ± 1.7	62.8 ± 2.4	62.4 ± 1.5	61.0 ± 2.0
	Tb.N (mm^{-1})	1.44 ± 0.17	1.43 ± 0.20	1.42 ± 0.15	1.37 ± 0.16
	Tb.Sp (mm)	0.57 ± 0.08	0.58 ± 0.08	0.58 ± 0.07	0.60 ± 0.08
	Tb.Th (mm)	0.134 ± 0.008	0.134 ± 0.010	0.131 ± 0.005	0.132 ± 0.005
**Humerus proximal**	BV/TV (%)	41.5 ± 2.0	45.0 ± 9.5	42.4 ± 1.8	42.9 ± 1.3
	Tb.N (mm^{-1})	3.49 ± 0.40	3.52 ± 0.35	3.73 ± 0.27	3.69 ± 0.46
	Tb.Sp (mm)	0.210 ± 0.032	0.200 ± 0.029	0.188 ± 0.020	0.193 ± 0.030
	Tb.Th (mm)	0.080 ± 0.003	0.087 ± 0.019	0.082 ± 0.001	0.081 ± 0.004
**Humerus distal**	BV/TV (%)	71.5 ± 2.1	71.3 ± 1.8	71.7 ± 2.1	70.8 ± 1.7
	Tb.N (mm^{-1})	3.59 ± 0.33	3.48 ± 0.33	3.38 ± 0.24	3.39 ± 0.30
	Tb.Sp (mm)	0.161 ± 0.025	0.171 ± 0.024	0.176 ± 0.017	0.179 ± 0.022
	Tb.Th (mm)	0.120 ± 0.004	0.119 ± 0.005	0.121 ± 0.006	0.118 ± 0.005
